# Sports Hernias: A Comprehensive Review for Clinicians

**DOI:** 10.7759/cureus.43283

**Published:** 2023-08-10

**Authors:** Michael Kopscik, Joseph L Crisman, Laurie Lomasney, Sean Smith, Shaheen Jadidi

**Affiliations:** 1 Family & Community Medicine, AnMed Health Medical Center, Anderson, USA; 2 Family & Community Medicine, Loyola University Medical Center, Chicago, USA; 3 Musculoskeletal Imaging, Interventional Radiology, Diagnostic Radiology, Loyola University Medical Center, Chicago, USA; 4 Musculoskeletal Radiology, Superior Sports Radiology, Greenville, USA; 5 Sports Medicine, Edward-Elmhurst Health/NorthShore University HealthSystem, Chicago, USA

**Keywords:** sports injuries, orthopedic sports medicine, groin pain, hockey groin syndrome, footballer’s groin, inguinal-related groin pain, inguinal disruption, gilmore groin, sportsman’s hernia, athletic pubalgia

## Abstract

Sports hernias are a complex cause of chronic groin pain in athletes, posing diagnostic and treatment challenges for clinicians. This review article synthesizes current knowledge on sports hernias, exploring pathogenesis, diagnostic approaches, and management strategies. Despite the growing body of research, sports hernias continue to present a significant challenge, necessitating a multidisciplinary approach and further research to improve clinical outcomes. This comprehensive review aims to equip clinicians with an updated understanding of sports hernias, ensuring optimal patient care and informing future research.

## Introduction and background

Sports hernias represent a complex and often under-recognized cause of chronic groin pain in athletes, which can significantly impair their performance and quality of life. Despite the myriad of terms used to describe this condition, the underlying pathophysiology of sports hernias remains elusive, as no single anatomical structure is consistently implicated in its development. This lack of consensus has led to diagnostic challenges and varying treatment approaches, making the management of sports hernias a topic of ongoing debate among clinicians [1].

In recent years, there has been an increased interest in understanding the etiology, risk factors, and biomechanics of sports hernias to improve diagnostic accuracy and tailor treatment strategies. The growing body of research has elucidated the multifactorial nature of this condition, implicating a combination of muscle imbalances, core instability, and pelvic dysfunction in the genesis of sports hernias. Moreover, the emerging role of advanced imaging modalities has shed light on the subtle anatomical changes that characterize these injuries, enabling clinicians to identify affected structures with greater precision [2].

The purpose of this comprehensive review is to provide clinicians with an updated understanding of sports hernias in the context of the latest advancements in the field. We will begin by delving into the current theories on pathogenesis, highlighting the diverse factors that contribute to the development of sports hernias. Next, we will explore the diagnostic process, focusing on the role of clinical examination, imaging studies, and the use of diagnostic algorithms to achieve a more accurate and timely diagnosis. Finally, we will discuss the management of sports hernias, examining both conservative and surgical approaches, as well as the role of rehabilitation in optimizing patient outcomes.

By synthesizing the most recent evidence on sports hernias, this review aims to equip clinicians with the knowledge and tools necessary to identify, diagnose, and manage these challenging injuries effectively, ultimately improving patient care and facilitating a timely return to sport.

## Review

Epidemiology

Understanding the epidemiology of sports hernias, also known as athletic pubalgia, is crucial for clinicians to effectively diagnose, manage, and prevent this condition. While the exact incidence of sports hernias remains unknown, certain trends in demographics, sports, and athletic levels have been identified [1]. There is a strong male predominance in the presentation of sports hernias, with males accounting for more than 90% of the cases [2]. This could be attributed to differences in pelvic anatomy, muscle strength, and/or the nature of the sports played by men, although more research is needed to confirm these hypotheses.

Regarding specific sports, sports hernias are most commonly observed in soccer players [3]. This is likely due to the dynamic nature of the sport, which involves rapid changes in direction, intense sprinting, and repetitive twisting and turning movements that place significant stress on the pelvic region. However, sports hernias have also been described in athletes participating in other sports such as football, hockey, baseball, and rugby [4]. These sports similarly involve high-intensity physical activity and maneuvers that can stress the muscles and soft tissues of the groin area, potentially leading to sports hernias.

Historically, sports hernias have been primarily diagnosed in professional or high-level athletes, who are subjected to more intense training regimens and have a higher likelihood of experiencing groin injuries. Nevertheless, in recent years, there has been a noted increase in the diagnosis of sports hernias among recreational athletes [5]. This trend may be attributed to a growing awareness of the condition, advancements in diagnostic techniques, and the increasing participation in recreational sports activities by individuals of all ages and fitness levels.

Etiology and pathophysiology

The etiology and pathophysiology of sports hernias, also known as athletic pubalgia, are not well understood, and the exact cause remains unclear. However, several factors have been proposed to contribute to the development of this condition, which will be discussed in this section.

The term “sports hernia” is a misnomer, as the majority of cases are not associated with tissue herniating through an identifiable fascial defect [6]. One hypothesis is that sports hernias may be related to overuse injuries in the context of underlying muscle imbalances. Specifically, an inequality in muscle strength, balance, stability, or endurance between a weaker abdominal musculature and a stronger hip adductor muscle group could lead to increased stress on the inguinal wall structures [7]. This imbalance can result in excessive strain on the inguinal region during athletic activities, predisposing an individual to the development of a sports hernia. In support of this theory, one study noted a deficient posterior wall of the inguinal canal in 85% of surgical cases involving sports hernias [8,9]. This posterior wall defect most commonly involved damage to the transversalis fascia, although pathology involving the conjoined tendon and internal inguinal ring was also noted [8]. This weakness is thought to be exacerbated by repetitive twisting, turning, or kicking movements, ultimately predisposing these individuals to a groin injury.

During surgery, multiple injured structures are often identified, which may provide insight into the complex pathophysiology of sports hernias. These injured structures can include the ones listed below [9].

Distal rectus abdominis: The distal portion of the rectus abdominis muscle, which is part of the abdominal musculature, may be subject to injury due to the aforementioned imbalances.

Conjoint tendon: This structure, which connects the internal oblique and transversus abdominis muscles to the pubic bone, can also be affected in sports hernia cases.

External oblique aponeurosis: The external oblique aponeurosis, a broad, flat tendon that connects the external oblique muscle to the pubic bone, can become damaged due to repetitive stresses from athletic activities. The ilioinguinal nerve runs adjacent to the external oblique aponeurosis before entering the external inguinal ring and has been similarly implicated in the symptomatology of sports hernias [10].

Inguinal ligament: The inguinal ligament, which forms the base of the inguinal canal, may also be compromised in cases of sports hernias. This ligament plays a crucial role in supporting the inguinal region and can be subject to injury when subjected to excessive stress.

Adductor longus muscle/tendon: The adductor longus, a hip adductor muscle that is often stronger in individuals with sports hernias, may also be injured due to the excessive forces placed on it during sports activities. This muscle injury could exacerbate the imbalance between the abdominal and hip adductor muscles, thereby increasing the risk of a sports hernia.

Matrix Metalloproteinases and Genetic Underpinnings

Although athletic pubalgia is a separate entity from classic inguinal hernias, the two might not be completely disparate in their underlying mechanisms. Recent studies are casting light on the shared foundations they might have, particularly concerning the structural soundness of the extracellular matrix and the possible role of genetics in predisposition. The structural integrity of the extracellular matrix is paramount for maintaining tissue resilience, especially in areas subjected to repetitive stresses, such as the inguinal region. A study spearheaded by the University of Naples Federico II has turned the spotlight on the role of matrix metalloproteinases (MMPs) in these vulnerabilities [11]. Elevated levels of MMPs in hernia-affected tissues hint at a potential weakening or compromise of the matrix, which might be instrumental in hernia onset. The interplay between these MMPs and their natural inhibitors, tissue inhibitors of metalloproteinases (TIMPs), is equally significant. An imbalance between MMPs and TIMPs could pave the way for structural vulnerabilities, making this axis a potential target of interest in understanding the pathophysiology of both inguinal hernias and athletic pubalgia.

Genetics seems to be playing its part in the broader conversation as well. A pioneering publication in Nature Communications unveiled specific genetic regions associated with hernia susceptibilities, emphasizing their quintessential roles in preserving the structural fidelity of connective tissues [12]. Notably, Genome-Wide Association Studies (GWAS) have been pivotal in deciphering these genetic markers, painting a clearer picture of how genetics might interplay with tissue vulnerabilities [13]. This begs an intriguing question: Are there unique genetic footprints that make certain individuals more susceptible to athletic pubalgia? The tapestry of research around inguinal hernias offers a repository of insights, many of which can be transposed to understand athletic pubalgia better. The dynamic between MMPs and TIMPs, coupled with the emerging genetic insights, might just be the tip of the iceberg. As research forges ahead, we can anticipate a future where our grasp of athletic pubalgia deepens, potentially leading to precise diagnostic tools, tailored therapeutic interventions, and preemptive measures for those in the crosshairs of sports hernias.

Risk factors and prevention

Understanding the risk factors and potential preventative measures for sports hernias is essential for clinicians to effectively manage and treat this condition. Athletic pubalgia occurs predominantly in sports that require sudden and forceful movements. Sports hernias have been observed to be particularly common in those involved in activities that demand sudden acceleration, deceleration, kicking, cutting, twisting, and turning. These activities place significant stress on the muscles, tendons, and ligaments in the groin and lower abdomen, increasing the likelihood of injury [14]. Sports with the highest risk of sports hernias include soccer, hockey, football, rugby, and track and field events such as sprinting and hurdling [15]. Athletes in these sports are particularly susceptible to sports hernias due to the frequent and intense demands on their lower abdominal and groin regions.

To minimize the risk of sports hernias, it is crucial to address the underlying factors that contribute to their development. Strength and conditioning programs that emphasize flexibility, core strength, and sport-specific movements have been suggested as possible preventative measures [16]. These programs should be tailored to the individual athlete, taking into account their specific sport, skill level, and physical condition. One potential contributing factor to sports hernias is a strength imbalance between the musculature below the pubic symphysis and the muscles above it. Strengthening the abdominal oblique and rectus muscles may help to correct this imbalance and, in turn, reduce the risk of injury [17]. However, it is important to note that research in this area is currently lacking, and more studies are needed to confirm the effectiveness of these targeted strengthening exercises in preventing sports hernias. In addition to targeted strength and conditioning programs, athletes should also focus on proper warm-up and cool-down routines, as well as sport-specific drills to improve their technique and movement efficiency [17]. Properly fitted and supportive footwear may also play a role in reducing the risk of injury by providing adequate stability and cushioning during high-impact activities.

History and presenting symptoms

The common history of present illness features in sports hernias or athletic pubalgia involves activity-related pain that is relieved with rest but returns upon resumption of sports. The pain typically has a gradual onset, worsens over time, and may affect daily activities. It is crucial for clinicians to consider these features when evaluating patients presenting with groin pain and to explore potential coexisting conditions to ensure a comprehensive assessment and appropriate treatment plan. Sports hernias typically present with a distinct set of symptoms related to the history of the patient’s illness, and the most common features include those listed below [7,10].

Activity-related pain: Affected individuals usually experience lower abdominal and proximal adductor-related pain during activities that involve quick acceleration, deceleration, kicking, twisting, or lateral movement. Soccer players, in particular, may develop sports hernias as a result of hard or long kicks.

Relief with rest: The pain associated with sports hernias tends to be relieved with rest but often returns once individuals resume their sport.

Gradual onset: Sports hernias can develop gradually over time, but they may also occur suddenly after a tearing sensation is felt during physical activity.

Pain progression: In the early stages of a sports hernia, pain is typically felt after the activity or toward the end of the activity. As the condition worsens, the pain becomes more severe and starts to occur earlier in the activity, leading to a decreased ability to twist, turn, or stride out. Eventually, the pain may be present during running and can progress to affect daily activities.

Pain distribution: Sports hernias are usually unilateral, but the pain may radiate to the uninvolved side or the scrotum.

Exacerbating factors: The pain associated with sports hernias can be reproduced by sudden movements, sit-ups, coughing, sneezing, and the Valsalva maneuver.

Hip joint-related mechanical symptoms: It is important for clinicians to inquire about any hip joint-related mechanical symptoms, such as catching, locking, and giving way, which may be indicative of underlying issues.

Physical examination features

The physical examination is a crucial component in the evaluation of sports hernias, also known as athletic pubalgia. A thorough examination helps in differentiating sports hernias from other causes of groin pain. The essential physical examination technique involves the inversion of the scrotal skin and palpation along the inguinal canal. A diagnosis of sports hernia may be made if at least three of the following five signs are present [9]: (1) Pinpoint tenderness to the pubic tubercle at the conjoint tendon insertion. (2) Tenderness over the deep inguinal ring. (3) Pain and/or dilation of the external ring without a palpable hernia. (4) Pain at the origin of the adductor longus tendon. (5) Dull, diffuse groin pain that often radiates to the perineum and inner thigh or across the midline.

In addition to these signs, a complete examination for other causes of groin pain should be performed: (1) Hip adductor origin tenderness and pain with resisted adduction suggest adductor-related groin pain. (2) Tenderness at the pubic symphysis indicates pubic-related groin pain. (3) Pain with resisted hip flexion and/or stretching of hip flexors suggests iliopsoas-related groin pain. (4) Hip joint-related groin pain may be identified by eliciting pain during passive range of motion (ROM) tests, such as flexion, adduction, and internal rotation (FADIR), as well as flexion, abduction, and external rotation (FABER) tests. (5) A genitourinary examination should be performed to rule out other potential causes of groin pain.

Differential diagnosis

When diagnosing sports hernias or athletic pubalgia, it is crucial for clinicians to consider a range of possible differential diagnoses. These conditions may present with similar symptoms, such as groin pain or discomfort, but require different management and treatment approaches. Some of the key differential diagnoses to consider include the ones listed below.

Inguinal or femoral hernia: Characterized by a palpable mass with appropriate maneuver, these hernias can cause groin pain similar to sports hernias. Physical examination techniques, such as the Valsalva maneuver, may help to identify these hernias.

Hip adductor strain [18]: Pain with resisted action of the adductor muscles may indicate a hip adductor strain, which can mimic sports hernia symptoms. Clinical assessment and imaging, such as ultrasound or magnetic resonance imaging (MRI), can help differentiate this condition from athletic pubalgia.

Rectus abdominis strain: This muscle strain in the abdomen can cause pain similar to sports hernias and may be confused with athletic pubalgia. A thorough clinical examination and imaging studies can help distinguish between the two conditions.

Femoroacetabular impingement (FAI) [19]: This condition, caused by abnormal contact between the femoral head and acetabulum, can lead to groin pain and may be mistaken for a sports hernia. Physical examination, including provocative tests, and imaging studies such as X-ray or MRI, can help identify FAI.

Osteitis pubis [20]: Inflammation of the pubic symphysis can cause chronic groin pain, which may be confused with athletic pubalgia. Diagnostic imaging, such as X-ray, MRI, or bone scintigraphy, can help differentiate osteitis pubis from sports hernias.

Bursitis: Inflammation of a bursa near the hip joint can cause pain that may mimic sports hernia symptoms. A thorough clinical examination and imaging studies can help identify bursitis and distinguish it from athletic pubalgia.

Snapping hip syndrome: This condition, characterized by a snapping or clicking sensation in the hip during movement, can cause groin pain and can be mistaken for a sports hernia. Physical examination, including assessment of the hip ROM and palpation of the hip joint, can help identify snapping hip syndrome.

Diagnostic workup and testing

Sports hernias can be challenging to diagnose due to the variety of potential causes and the often nonspecific nature of symptoms. In addition, athletic pubalgia often coexists with other causes of chronic groin pain [16]. While a thorough physical examination remains crucial for the diagnosis of sports hernias and should always be performed, the role of additional diagnostic tests will be discussed in this section. It is important to note that athletic pubalgia is a clinical diagnosis, and cannot be ruled out by any single imaging study [21]. This section will provide a comprehensive overview of the diagnostic tests and interpretations used to identify sports hernias and related conditions. Most patients with this condition will have no pathologic imaging findings. However, these tests can still be helpful in ruling out other potential causes of groin pain.

Magnetic Resonance Imaging

**Figure 1:**
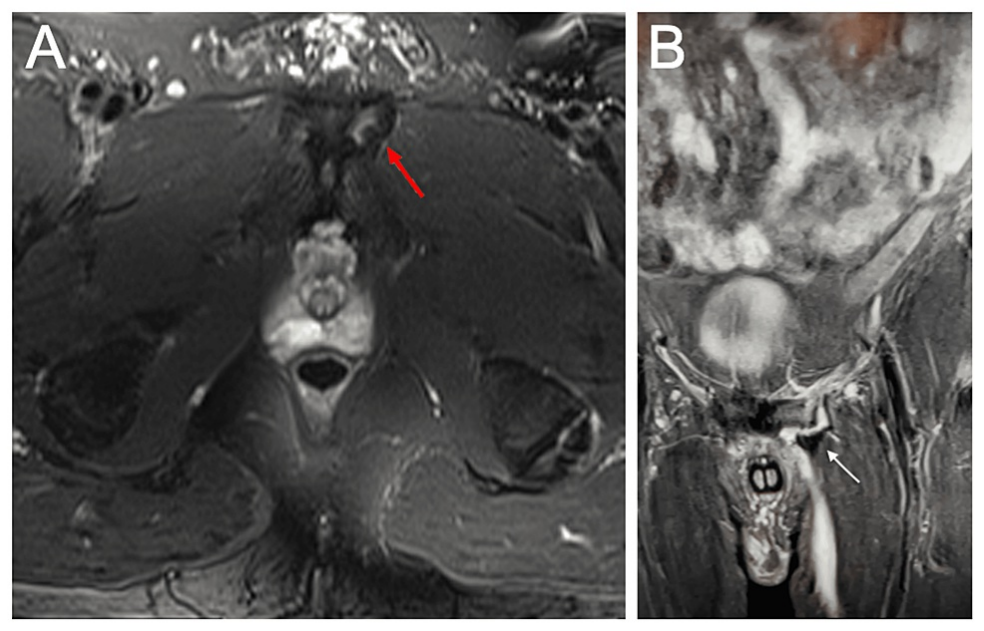
Axial (A) and coronal (B) T2 fat saturation MRI images through the symphysis pubis. A: T2 axial imaging displays an amplified signal within and around the anterosuperior segment of the pubic body (red arrow). This signal is discernible directly under the insertion point of the common adductor-rectus abdominis aponeurosis, indicating long-term tendon and bone interface disruption. B: Tear of the adductor longus muscle tendon (white arrow) from the symphysis with slight retraction and fluid (white signal) between the tendon and bone.

MRI is the most sensitive imaging modality to evaluate for sports hernias. Specific patterns of injury have been identified, including tears of the rectus abdominis and adductor aponeurosis [7] as well as erosion and/or osteitis of the pubic bodies (Figure 1). However, these findings are not specific to athletic pubalgia. Similar to radiographs, MRI is often used to exclude alternative causes of chronic groin pain due to its high sensitivity in identifying injury to adjacent structures [9].

When ordering an MRI for a suspected sports hernia, clinicians should request a dedicated pelvis MRI with attention to the pubic symphysis and surrounding soft tissue structures. This imaging protocol should include T1-weighted and T2-weighted (including fat saturation) sequences in multiple planes. Axial and oblique axial as well as coronal planes are most contributory. The use of intravenous contrast may be beneficial in cases with subtle imaging findings to better visualize any inflammation or injury to the involved structures [22]. To assure focused imaging, clinicians must communicate their specific clinical suspicion for a sports hernia to the radiologist, as this will likely influence protocol selection as well as the radiologist’s interpretation of the images.

Dynamic Ultrasound

Dynamic ultrasound is increasingly utilized in the evaluation of posterior inguinal wall insufficiency, which is commonly seen in sports hernias. This noninvasive imaging modality offers several advantages, such as real-time assessment, minimal discomfort, and the ability to evaluate structures during different phases of movement. However, the presence of posterior inguinal wall insufficiency has low sensitivity and specificity for diagnosing sports hernias, as it may also be observed in asymptomatic athletes [23]. Furthermore, ultrasound is highly operator dependent and has limited availability. During an ultrasound examination, a skilled sonographer can identify various features associated with sports hernias. These features include soft tissue abnormalities such as anechoic defects and/or partial or full-thickness discontinuities of the tendon insertions, indicative of tears; loss of normal fibrillary architecture, thickening, and hypoechogenicity of tendon insertions, suggestive of tendinopathy. Bone changes may include spurring and irregularities of the pubic bone, indicative of chronic stress or injury; symphyseal effusion, representing joint inflammation or injury; and periarticular hyperemia on color Doppler, revealing increased blood flow around the affected area. Reproduction of symptoms on the pressure with the ultrasound transducer is especially valuable to confirm the location of pain or discomfort [24]. However, there are potential pitfalls in the ultrasound assessment for sports hernias. Anisotropy, an artifact that occurs when the angle of the ultrasound beam relative to the tendon fibers changes, can result in the false appearance of tendinosis. Correct positioning of the transducer and using appropriate settings can help minimize this artifact. Additionally, protrusion of pre-peritoneal fat through the Hesselbach triangle during abdominal strain can mimic the appearance of a hernia. Recognizing this finding and differentiating it from a true sports hernia is crucial for accurate diagnosis.

Radiographs

Diagnostic workup for sports hernias often involves the use of radiographs as a first-line imaging modality in the sports medicine clinic. These imaging techniques can provide valuable insights into the underlying causes of groin pain, helping physicians to diagnose or exclude sports hernias and other conditions that might contribute to discomfort in the region [25]. For example, radiographs may reveal alternative diagnoses including fractures, degenerative disease, FAI, dysplasia, and pubic symphysis asymmetry [26]. Additionally, alignment abnormalities, such as pelvic tilt or leg length discrepancies that can contribute to groin pain and muscular imbalances, may be identified on standing radiographs.

Computed Tomography Scan

Computed tomography (CT) scans can provide more detailed information on bony structures and soft tissues surrounding the groin area. These scans can help identify the widening of the symphysis pubis, a potential indicator of pubic instability or stress injuries, which may contribute to the development of sports hernias or cause similar symptoms to a sports hernia. Additional findings of reactive sclerosis, marginal erosion, and/or capsular thickening are supportive findings. CT scans can also assist in excluding other causes of groin pain, such as osteoarthritis or FAI, by providing a more precise analysis of joint spaces, cartilage, and bony morphology [27,28]. Identifying these conditions may help guide appropriate treatment and prevent unnecessary interventions for a sports hernia.

Herniography

Herniography is an invasive diagnostic test that involves the injection of contrast material into the peritoneal cavity to visualize the inguinal region. Although highly sensitive and specific at diagnosing “true” hernias, herniography is generally not recommended as a first-line diagnostic tool for sports hernias due to its invasive nature [29].

Nuclear Medicine Bone Scintigraphy

Nuclear medicine bone scan is not typically indicated for the diagnosis of athletic pubalgia. Although this modality is exquisitely sensitive to the pathology of the bony skeleton and readily detects fractures, osteoarthritis, and osteomyelitis, the resolution is insufficient to differentiate reactive osteitis and fracture, for example. Further, the lack of soft tissue analysis precludes the evaluation of marginal tendons and the aponeurotic condensation, anatomic structures that must be evaluated in this clinical setting [30].

Treatment

General Measures

Nonoperative treatment should be attempted before surgical consideration, as conservative management is often successful in managing sports hernias. This typically includes a nonsurgical rehabilitation treatment plan consisting of an initial six- to eight-week period of rest with supportive care, such as nonsteroidal anti-inflammatory drugs, ice, and heat [7,31]. After this period, a six-week rehabilitation program with a gradual return to sports activity is recommended [31].

The rehabilitation program should initially focus on hip adductor stretching and core stabilization exercises, before advancing to eccentric strengthening of abdominal oblique, rectus abdominis, and adductors [16,17]. Finally, the program should progress to sports-specific functional exercises. The entire process may take as long as two to three months. Ultrasound-guided injection to rectus abdominis insertion, conjoint tendon, or adductor tendon may be considered, but there is limited evidence for its efficacy [17].

Surgery/Other Procedures

Surgery should be considered if nonoperative treatment fails after three or more months [17]. Herniorrhaphy is the definitive treatment for sports hernias. Although surgical techniques vary, the general principles are to release abnormal tension on the inguinal canal and reinforce the posterior wall of the inguinal canal [32]. There are two main types of surgery for sports hernias, namely, open and laparoscopic techniques.

Open surgery involves a larger incision, and while it has been reported to be effective, it may be associated with a longer recovery time compared to laparoscopic surgery [33]. Laparoscopic surgery, on the other hand, involves smaller incisions and allows for a quicker return to sport [34]. Both open and laparoscopic surgical techniques have been reported to be effective at similar rates [17].

Return to Sport

Returning to sport following treatment for athletic pubalgia, whether pursued through conservative or surgical avenues, is shaped by the treatment’s modality, individualized factors, and injury severity. The time to fully return to sports ranges from four to 12 weeks depending on the type of surgery and individual recovery factors [32]. Post-surgery, the rate of return to the prior level of activity is reported to be between 80% and 95% [35,36]. To optimize surgical outcomes, it is essential that clinicians and patients carefully consider the most appropriate surgical approach based on the patient’s specific condition, preferences, and goals.

Trends drawn from recent literature suggest that return-to-play (RTP) timelines, while variable, lean toward promising outcomes. For instance, in a surgical approach tailored for athletic pubalgia by Kajetanek and colleagues, an impressive 92.6% of subjects made a successful RTP to their pre-injury sport levels within an average span of 112 ± 38 days. It is noteworthy that athletes with sole lower abdominal wall injuries reported a swifter recovery, clocking in at about 91.1 ± 21.0 days, particularly when contrasted with their counterparts who sustained only adductor tendon injuries or a combination of injuries [37]. Diving deeper into the realm of professional sports, Castle et al. identified that a substantial 90.91% of NBA players resume play after their surgical intervention for athletic pubalgia, albeit within a slightly protracted average timeline of 4.73 ± 2.62 months. However, a caveat looms over this statistic; these athletes have been observed to experience a contraction in their career longevity subsequent to the surgery [38]. Expanding the scope, Serafim et al., through a comprehensive review, inferred that athletes opting for surgery often rejoin their sport sooner than those adopting conservative treatments. Yet, they underscore the primacy of weighing conservative strategies before leaping to surgical decisions [39].

Mirroring investigations and discourses from other athletic milieus, the overarching narrative points toward commendable RTP rates, often transcending the 90% mark, post-treatment for athletic pubalgia. It is imperative, however, to not merely assess RTP in isolation but to juxtapose it with athletes’ capacities to revert to their pre-injury prowess and gauge it against their contemporaries. Despite occasional discrepancies in the data regarding post-surgical game involvement and proficiency in professional sports spheres, a consensus is building around the optimistic potential for athletes to retrace their path back to full-fledged gameplay and performance, especially in the aftermath of surgical intervention for athletic pubalgia.

## Conclusions

Sports hernias continue to present a significant challenge for clinicians due to their complex and multifaceted nature. Accurate diagnosis and effective treatment of sportsman’s hernia necessitate a careful clinical evaluation, a multidisciplinary approach, and experience. As the response rates to conservative treatment for chronic inguinal pain are disappointingly low, it is crucial for healthcare professionals to consider alternative options when appropriate.

A deeper understanding of the pathophysiology of sports hernias is essential for the improvement of clinical outcomes and the reduction of remission periods. Further research is required to elucidate the underlying mechanisms and develop targeted therapeutic interventions. Additionally, prospective randomized studies focusing on specific surgical techniques are needed to provide more robust evidence and guidance for clinicians, ultimately helping to resolve ongoing debates surrounding treatment options.

Sports hernias demand a comprehensive and evidence-based approach to ensure optimal patient care. As our understanding of sportsman’s hernia continues to evolve, it is essential for clinicians to stay up-to-date with the latest research and advancements in this field to provide the best possible outcomes for their patients.
